# The Adjustment of Membrane Lipid Metabolism Pathways in Maize Roots Under Saline–Alkaline Stress

**DOI:** 10.3389/fpls.2021.635327

**Published:** 2021-03-15

**Authors:** Xiaoxuan Xu, Jinjie Zhang, Bowei Yan, Yulei Wei, Shengnan Ge, Jiaxin Li, Yu Han, Zuotong Li, Changjiang Zhao, Jingyu Xu

**Affiliations:** ^1^Key Lab of Modern Agricultural Cultivation and Crop Germplasm Improvement of Heilongjiang Province, Heilongjiang Engineering Technology Research Center for Crop Straw Utilization, College of Agriculture, Heilongjiang Bayi Agricultural University, Daqing, China; ^2^Beijing Hortipolaris Co., Ltd., Beijing, China; ^3^Institute of Industrial Crops, Heilongjiang Academy of Agricultural Sciences, Harbin, China

**Keywords:** maize (*Zea mays*), lipid metabolism, lipidome, transcriptome, saline–alkaline stress

## Abstract

Plants are frequently confronted by diverse environmental stress, and the membrane lipids remodeling and signaling are essential for modulating the stress responses. Saline–alkaline stress is a major osmotic stress affecting the growth and development of crops. In this study, an integrated transcriptomic and lipidomic analysis was performed, and the metabolic changes of membrane lipid metabolism in maize (*Zea mays*) roots under saline–alkaline stress were investigated. The results revealed that phospholipids were major membrane lipids in maize roots, and phosphatidylcholine (PC) accounts for approximately 40% of the total lipids. Under 100 mmol NaHCO_3_ treatment, the level of PC decreased significantly (11–16%) and the parallel transcriptomic analysis showed an increased expression of genes encoding phospholipase A and phospholipase D/non-specific phospholipase C, which suggested an activated PC turnover under saline–alkaline stress. The plastidic galactolipid synthesis was also activated, and an abnormal generation of C34:6 galactolipids in 18:3 plants maize implied a plausible contribution from the prokaryotic pathway, which could be partially supported by the up-regulated expression of three putative plastid-localized phosphatidic acid phosphatase/lipid phosphate phosphatase. A comprehensive gene–metabolite network was constructed, and the regulation of membrane lipid metabolism under saline–alkaline stress in maize was discussed.

## Introduction

Cell membrane is the first parclose of defense for plants to cope with the external environmental stimuli, and its fluidity and stability are critical for the survival of cells and even whole plants under various stress conditions (Sulian et al., 2020). In plants and other organisms, the structure and properties of membrane are determined by a complex mixture of lipid species with different head group structures, fatty acid (FA) lengths, and unsaturation (Mikami and Murata, 2003; Li N. et al., 2016).

Plasma membranes of plant cells are mainly composed of glycerolipids. Phospholipids dominate the extraplastidic glycerolipids, including phosphatidylcholine (PC), phosphatidylethanolamine (PE), phosphatidic acid (PA), phosphatidylinositol (PI), and phosphatidylserine (PS), whereas the major components of the plastidic membrane are monogalactosyldiacylglycerol (MGDG), digalactosyldiacylglycerol (DGDG), phosphatidyl glycerol (PG), and sulfoquinovosyl diacylglycerol (SQDG) (Benning, 2009). Studies have shown that the physical properties of the membrane can be altered by changing the relative amount of each lipid class (Botella et al., 2017). The metabolic changes of membrane lipids are closely related to the stress responses of plant, and frequent lipid remodeling has been observed in membrane lipids under various stress conditions (Gu et al., 2017). Narasimhan et al. (2013) found that the increase of PA would affect root growth and proliferation through its effect on vesicle transport and cytoskeletal recombination under the condition of P and N defects. Studies have been shown that phospholipase D (PLD) and non-specific phospholipase C (NPC), which direct dephosphorylation of endoplasmic reticulum (ER) PC into PA and diacylglycerol (DAG), respectively, involved in lipid remodeling (Nakamura et al., 2009).

The synthesis of glyceroglycolipids has two different pathways, which are compartmentalized in two different subcellular spaces and work synergistically (Li-Beisson et al., 2013; Li Q. et al., 2016). One pathway is the eukaryotic pathway, also known as the ER pathway, which is implemented in the ER, and the other is the prokaryotic pathway, also known as the plastidic pathway, which is localized in the plastid envelope (Li-Beisson et al., 2013). Numerous previous studies have shown that the balance of prokaryotic and eukaryotic pathways of lipid synthesis in *Arabidopsis* is associated with abiotic stress, and the contribution of the enhanced eukaryotic pathway to lipid synthesis can be observed under various abiotic stress conditions (Nakamura et al., 2009; Moellering and Benning, 2011; Higashi et al., 2015; Li et al., 2015). Previous biochemical and molecular biology studies have shown the synergistic regulation of the key enzymes involved in these two pathways (Shen et al., 2010). In *Arabidopsis*, light and temperature stimulation promotes their interaction, and marked changes in FA molecular species of the membrane lipids occurred (Burgos et al., 2011; Szymanski et al., 2014; Li et al., 2015). In maize (*Zea mays*), low temperature stress induced the expression of lipid related genes in the eukaryotic pathway and led to enhanced flow of lipids products to plastids/chloroplasts (Gu et al., 2017).

Saline–alkaline stress is a major osmotic stress affecting the growth and development of crops. Previous study revealed that salinity could induce membrane structure and lipid changes in maize mesophyll and bundle sheath chloroplasts (Omoto et al., 2016). In this study, the combined analysis of transcriptome and lipidome was conducted to investigate the transcriptional regulation of lipid metabolism in the root of maize under saline–alkaline stress and to provide a better understanding of saline–alkaline stress response mechanism in major field crops.

## Materials and Methods

### Plant Treatments and Sampling

The seeds of inbred maize variety He 344 were selected and disinfected with 1% sodium hypochlorite for 30 min and then were washed with tap water and rinsed with distilled water for three times. The seeds were soaked in nutrient solution 6–8 h for germination. When the buds were 0.5 cm long, they were placed in perforated plates and cultured in 1/2 Hoagland nutrient solution (LEAGEN) in an artificial chamber (KUANSONS, 22°C, 16 h light/8-h dark, 250 μmol m^–2^ s^–1^ photosynthetic photon flux density and 60–65% relative humidity). The maize seedlings grown to the “two leaves and one heart” stage (around 2 weeks old) were treated with 1/2 Hoagland nutrient solution supplemented with 100 mmol NaHCO_3_, and the control group was left in the 1/2 Hoagland nutrient solution, and then the root tissues of maize seedlings were sampled at 24 and 72 h after treatment, respectively.

### Transcriptomic Analysis and Quantitative Reverse Transcriptase–Polymerase Chain Reaction Validation

The total RNA of the maize root tissues was extracted using TRIZOL reagent (Invitrogen). The construction of cDNA library was started from the synthesis of two cDNA strands, followed by the purification of double-stranded cDNA and the enrichment of cDNA library by polymerase chain reaction (PCR) amplification (TOYOBO). The Illumina HiSeq 2000 platform was used for the RNA-seq sequencing of the constructed library (BioMarker). The obtained unigene sequences were aligned with the protein databases NR, NT, SwissProt, KEGG, COG, and GO using Blast software^1^ (*E*-value < 1.0E^–5^) for the best feature annotation. The differentially expressed genes (DEGs) were determined, and the significant DEGs were further screened out based on the criteria of Log_2_FC ≥ 1.5 or ≤ -1.5 (false discovery rate ≤ 0.01). The RNA-seq data have been submitted to the online SRA (sequential read file) database with accession number SRP307694.

Quantitative reverse transcriptase (RT)–PCR analysis was performed to verify the validity of RNA-seq data. The gene primers were designed by Primer 5 software. Maize *actin* (*GRMZM2G126010*) and *GAPDH* (*GRMZM2G155348*) genes were used as internal controls. The extracted RNA samples were reverse transcribed according to the instructions of ReverTra Ace qPCR RT Master Mix with gDNA remover reverse transcription kit (TOYOBO). Fluorescence quantitative PCR reactions were performed according to the instructions of THUNDERBIRD SYBR qPCR mixture real-time quantification kit (TOYOBO). Each reaction was repeated three times, and three biological replicates were set up for each sample (see Supplementary Figure S1 for details).

### Membrane Lipid Extraction and Analysis

After treatment with NaHCO_3_ (100 mmol) at different time points, root samples from 2 week-old maize seedlings were collected from five plants in different pots (samples from plants without NaHCO_3_ treatment were used as control), and the lipidomic analysis was conducted in six replicates. The extraction of total plant lipids was modified based on previous reports (Narayanan et al., 2016). Approximately 200 mg of fresh root tissues was quickly immersed in a 50 mL glass tube (Teflon lining, threaded cap) containing 3 mL of 0.01% BHT (SIGMA) in hot isopropanol solution (75°C). After cooling to room temperature, 1.5 mL of chloroform and 0.6 mL of water were added and vortexed for 1 h. The extract was then placed in a new tube, and 4 mL of chloroform/methanol (2:1) containing 0.01% BHT was added. The mixture was shaken for 30 min, and the extraction process was repeated several times until the tissues turned white. One milliliter of 1 M KCl was added to the mixed extract, and then the new mixture was centrifuged, and the supernatant was discarded. The extract was washed again with 2 mL of water, and supernatant was discarded, and the remaining lipid extract was fully vaporized under a nitrogen blower. The dried lipid samples were stored at −80°C.

For FA compositional analyses, the extracts were dissolved in 1 mL of chloroform and precise amounts of internal standards, obtained and quantified as previously described (Welti et al., 2002). Unfractionated lipid extracts were introduced by continuous infusion into the Essential Science Indicators (ESI) source on a triple quadrupole MS/MS (API4000, ABSciex, Framingham, MA, United States). Samples were introduced using an autosampler (LCMini PAL, CTC Analytics AG, Zwingen, Switzerland) fitted with the required injection loop for the acquisition time and presented to the ESI needle at 30 μL min^–1^ (Narayanan et al., 2016). The precursor and neutral loss scans were applied to obtain polar lipid profiles. The sample is introduced into the electrospray ionization source, further generation of lipid molecular ions, including PC-, lysoPC-, PE-, and lysoPE-positive [M + H]^+^ ions; MGDG-, DGDG-, PG-, PI-, PA-, and PS-positive [M + NH_4_]^+^ ions; and lysoPG-negative [M-H]^–^ ions. A series of peak values of lipid content were detected by electrospray ionization. The peaks on the spectra are quantified in comparison to a group of internal standards. Mass spectrometry lipid analysis was performed at the Kansas Lipidomics Research Center (KLRC, United States) using electrospray ionization mass spectrometry.

The data for each lipid molecular species were normalized and displayed as mol% of the total lipids analyzed. Lipidomics results were expressed as values (mol%) = mean 5 ± standard deviation, three biological replicates for each set of data, and statistical analysis was performed by SPSS Statistic 21.0 with a significant level set to *a* = 0.05.

### Transmembrane Structure and Subcellular Location Analysis

The analysis of the structural domain of the maize ZmPAPs was through the Phytozome v13.0 database and the SMART website, and the transmembrane structure information of the maize ZmPAP gene was obtained through the Expasy online prediction website^2^. The protein sequences of ZmPAPs were submitted to WOLF PORST^3^ to predict the subcellular location of the ZmPAP proteins.

### Statistical Analysis

All statistical analyses were conducted with SPSS statistics 21.0 (SPSS Inc.). Five to six biological replicates for lipidomic data and three biological replicates for gene expression data were subjected for the calculation. The significance levels were calculated using Student *t*-test method. ^∗^*P* < 0.05 and ^∗∗^*P* < 0.01 represent difference significance levels.

## Results

### Lipids Metabolic Changes in Maize Roots Under Saline–Alkaline Stress

To investigate the changes of membrane lipids in maize root under NaHCO_3_ treatment (100 mmol), lipidomic analysis was conducted to analyze the various glycerolipids content and their FA composition. A total of 11 glycerolipids were detected by ESI-MS/MS, including six types of phospholipids (PC, PE, PA, PI, PS, and PG), two types of galactolipids (MGDG and DGDG), and three classes of lysophospholipids (LPG, LPC, and LPA).

**FIGURE 1:**
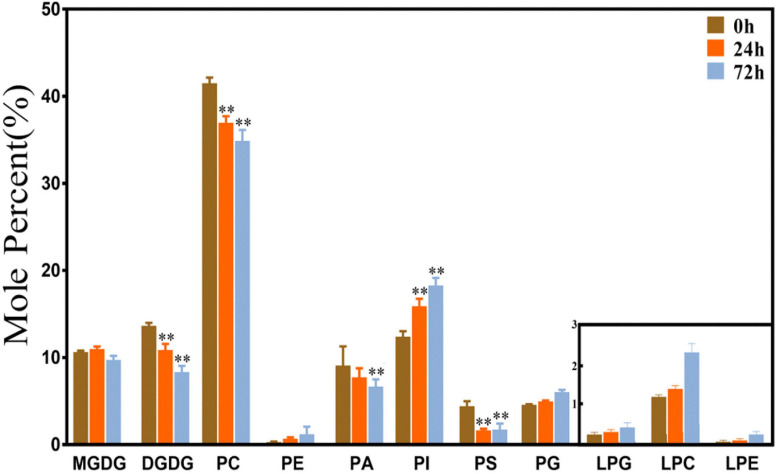
Changes of glycerolipids species in maize roots under NaHCO_3_ (100 mmol). MGDG, monogalactosyldiacylglycerol; DGDG, digalactosyldiacylglycerol; PC, phosphatidylcholine; PE, phosphatidylethanolamine; PA, phosphatidic acid; PI, phosphatidylinositol; PS, phosphatidylserine. PG, phosphatidylglycerol; LPG, Lyso-PC; LPC, Lyso-PC; LPA, Lyso-PC. Values (mol%) are means 5 ± standard deviation (SD), three biological replicates for each set of data. “**” indicated that the value was significantly different from the control (*P* < 0.01).

As shown in Figure 1, phospholipids are the main membrane lipids in maize root tissues, which account for approximately 70% of the total components. PC was the most abundant lipid species, accounting for more than 40% of all lipids, and the remaining phospholipid species account for another 30%. The proportion of the galactolipids MGDG and DGDG is approximately 20% of the total membrane lipids, with each accounting for 10%, respectively. The levels of three lysophospholipids (LPG, LPC, and LPA) were relatively low. Under saline–alkaline stress (100 mmol NaHCO_3_ treatment), the molar percentage of most phospholipid species was declined. The level of PC decreased around 11 and 16% after 24 and 72 h treatment, respectively, in comparison with the control. PA and PS also decreased in various degrees. The reduction of PA is 15 and 26% at 24 and 72 h time points, respectively, and PS decreased more than 50% at both time points. In contrast, the level of PI was elevated about 1.3–1.5 times under NaHCO_3_ treatment. The level of the plastidic phospholipid PG was increased, especially at the 72 h time point, which was 30% higher than the control. Regarding the galactolipids, the percentage of DGDG decreased significantly under NaHCO_3_ stress, which reduced 20 and 39% at 24 and 72 h, respectively. Although the content of lysophospholipids was low, the levels of LPG, LPC, and LPE were observed to be increased, and LPC showed a significant increase at the 72 h time point.

### The Changes of Fatty Acid Molecular Species Under Saline–Alkaline Stress

**FIGURE 2:**
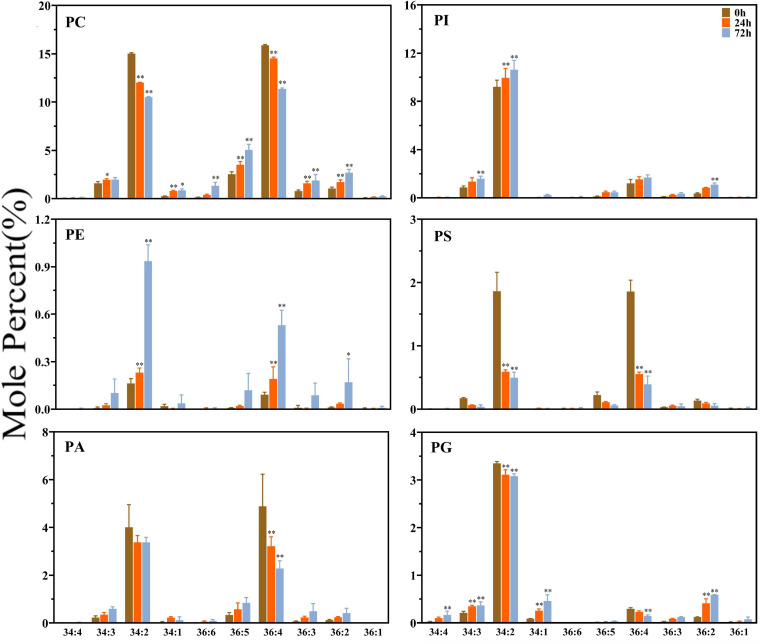
Changes in diacyl lipid molecular species of major phospholipids in maize roots under NaHCO_3_ (100 mmol). Major phospholipids: PC, phosphatidylcholine; PE, phosphatidylethanolamine; PA, phosphatidic acid; PI, phosphatidylinositol; PS, phosphatidylserine. PG, phosphatidylglycerol. Values (mol%) are means 5 ± standard deviation (SD), three biological replicates for each set of data. “*” indicated that the value was significantly different from the control (*P* < 0.05). “**” indicated that the value was significantly different from the control (*P* < 0.01).

The profiles of the FA molecules of the two side chains of the glycerolipids were also determined by lipidomic analysis. The molecular species are represented by the total number of carbon atoms vs. the total number of double bonds. As shown in Figure 2, the glycerolipids in maize root tissues have distinct FA profiles, which are particularly enriched in C34 and C36 molecules (the total number of acyl carbon atoms), and relatively higher molar percentage of C34:2 and C36:4 (the total number of acyl carbon atoms: double bonds) was observed. Most of the extraplastidic phospholipids classes (PC, PE, PA, and PS) were composed of similar proportion of C36 and C34 molecular species, whereas in the plastidic phospholipid PG, C34 molecules (especially C34:2) were dominant. Under saline–alkaline stress (100 mmol NaHCO_3_ treatment), the molar percentage of C34:2 and C36:4 molecules in the major phospholipids (PC, PA, PS, and PG) were decreased, whereas the percentage of C36:2, C36:5, and C34:3 was increased to varied degrees.

**FIGURE 3:**
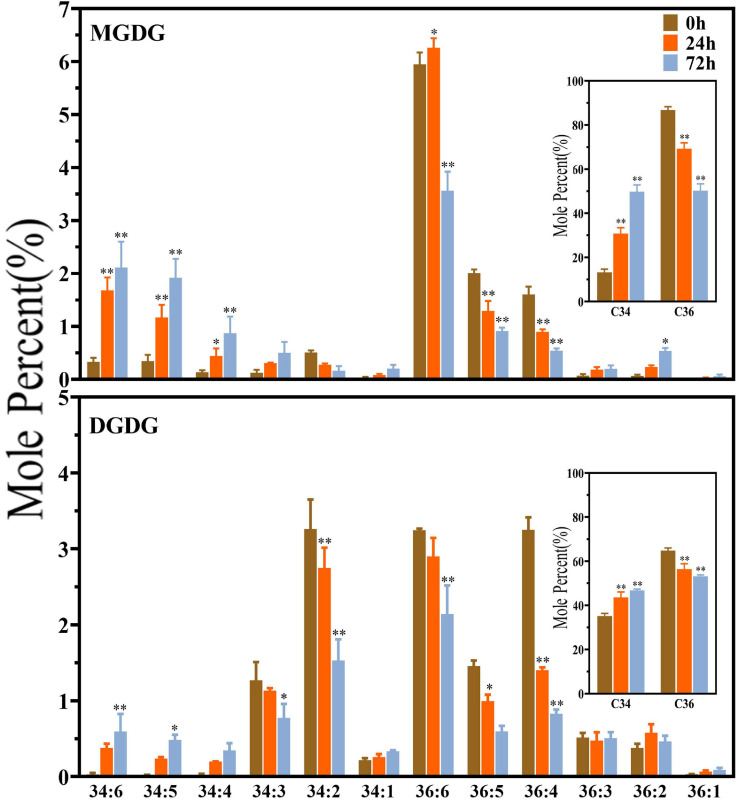
Changes in diacyl lipid molecular species of major plastidic galactolipids in maize roots under NaHCO_3_ (100 mmol). MGDG, monogalactosyldiacylglycerol; DGDG, digalactosyldiacylglycerol. Values (mol%) are means 5 ± standard deviation (SD), three biological replicates for each set of data. “*” indicated that the value was significantly different from the control (*P* < 0.05). “**” indicated that the value was significantly different from the control (*P* < 0.01).

As shown in Figure 3, the plastidic galactolipids MGDG and DGDG were mainly composed of C34 and C36 molecules, and C36 molecules were obviously dominant in MGDG. Under saline–alkaline stress (100 mmol NaHCO_3_ treatment), the molar percentage of C36 molecules decreased, whereas that of C34 molecules increased significantly in both MGDG and DGDG. In MGDG, the level of C34 was around two times higher than that of control after 24 h of NaHCO_3_ treatment and approximately three times higher after 72 h treatment in comparison with the control. It is worth noting that the level of polyunsaturated C34 molecules, including that of C34:4, C34:5, C34:6, was found tremendously elevated under NaHCO_3_ treatment (3–6-fold). In DGDG, the increase in the level of C34:4, C34:5, C34:6 under saline–alkaline stress was also obvious.

### Transcriptomic Analysis of Maize Roots Under Saline–Alkaline Stress

In order to investigate the molecular regulation of lipid metabolism in maize root tissues, the transcriptome was analyzed by means of Illumina RNA-seq. RNA was prepared from maize root samples collected from 2 week-old seedlings, after treatment with 100 mmol NaHCO_3_ for 0 (control), 24 and 72 h. Real-time PCR analysis was performed on a number of DEGs to validate the RNA-seq data. The results showed that all six tested genes exhibited similar expression profiles, which proved the reliability of the transcriptome data (Supplementary Figure S1). The RNA-seq data have been submitted to the online SRA database with an accession number of SRP307694.

**FIGURE 4:**
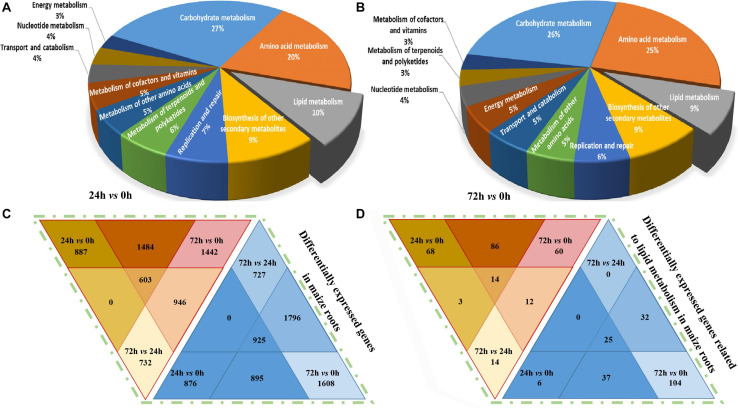
KEGG classification and DEGs (Log_2_FC ≥ 1.5 or ≤ -1.5) distribution analysis in maize roots transcriptome under NaHCO_3_ (100 mmol). **(A,B)** Kyoto Encyclopedia of Genes and Genomes (KEGG) pathway analysis with pie chart showing the distribution of metabolism-related differentially expressed genes (DEGs) identified in the maize roots’ transcriptome. **(A)** 24 h vs. 0 h, **(B)** 72 h vs. 0 h. **(C,D)** Venn diagram showing differentially expressed genes in maize roots transcriptome. **(C)** Differentially expressed genes in maize. **(D)** Differentially expressed genes related to lipid metabolism in maize roots. The color scale indicates the expression value: red indicates high level; blue indicates low level.

The obtained unigene sequences were aligned with the NR, NT, SwissProt, KEGG, COG, and GO protein databases. As shown in Figure 4A, in the “24 h vs. 0 h” comparison group, a total of 4,761 DEGs (Log_2_FC ≥ 1.5 or ≤ -1.5) were annotated and characterized into 136 metabolic pathways, among which the “carbohydrate metabolism,” “amino acid metabolism,” and “lipid metabolism” are the three most enriched metabolic pathways, and the lipid metabolism-related DEGs accounted for 10% in total DEGs. In the “72 h vs. 0 h” comparison group (Figure 4B), a total of 5,623 DEGs associated with 153 metabolic pathways were annotated, and approximately 9% of which are involved in lipid metabolism pathways.

The DEGs were determined, and their differential enrichment in different comparison groups is analyzed. As shown in Figure 4C, a total of 26,435 up-regulated genes and 29,048 down-regulated genes were obtained in the entire transcriptome. In the “24 h vs. 0 h” comparison group, there were 8,887 up-regulated genes and 7,166 down-regulated genes. In the “72 h vs. 0 h” group, the largest number of DEGs was obtained, and the up-regulated genes and down-regulated genes were 10,416 and 12,215, respectively. In the “72 h vs. 24 h” group, there were 7,132 up-regulated genes and 9,667 down-regulated genes, respectively. In general, the down-regulated DEGs in maize roots under saline–alkaline stress were slightly more than the up-regulated ones. In the three-group joint analysis, the number of the up-regulated DEGs was 603, which was 322 fewer than the 925 down-regulated DEGs. We also compared the distribution of DEGs related to lipid metabolism, and 387 up-regulated DEGs and 621 down-regulated genes were annotated in the transcriptome data, and their distribution was consistent with the overall trend of the transcriptome (Figure 4D). In the “24 h vs. 0 h” comparison group, 171 genes were up-regulated, and 68 genes were down-regulated. In the “72 h vs. 0 h” group, 173 genes were found up-regulated and 197 genes were down-regulated. In the “72 h vs. 24 h” group, 43 genes were up-regulated, and 356 genes were down-regulated.

### Differential Responses of Lipid Metabolism and Signaling Pathways in Maize Roots Under Saline–Alkaline Stress

**FIGURE 5:**
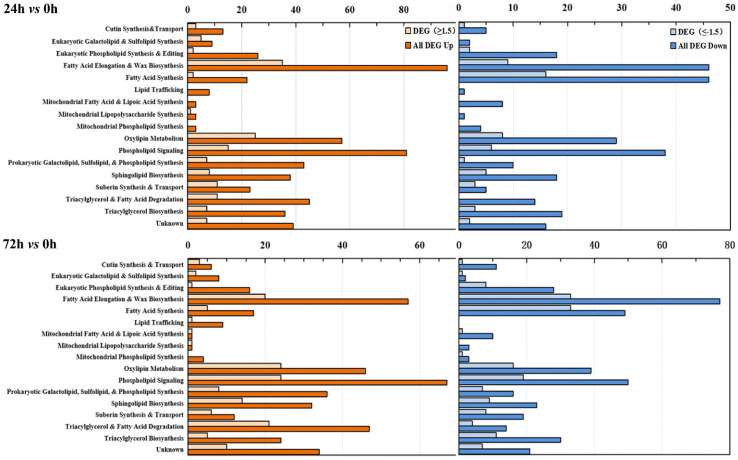
Functional categorization of lipid-related genes from maize roots transcriptome of under NaHCO_3_ stress (100 mmol). Genes involved in lipid metabolism were compiled from the *Arabidopsis* lipid gene database (Beisson et al., 2003) and categorized into different pathways. The upper panel is “24 h vs. 0 h,” and the lower panel is “72 h vs. 0 h.” Yellow columns represent down-regulated genes, and blue columns represent up-regulated genes. In each category, the light-colored column represents the significantly differentially expressed genes (DEGs, Log_2_FC ≥ 1.5 or ≤ -1.5), and the dark-colored column represents the total differentially expressed genes (DEGs). The number of the genes in each category was displayed in the *x*-axis (the ones on the upper *x*-axis indicate the DEGs, Log_2_FC ≥ 1.5 or ≤ -1.5).

Based on previously published *Arabidopsis* lipid gene database and related literature (Li-Beisson et al., 2013; Troncoso-Ponce et al., 2013; Botella et al., 2017), and combined with GO and KEGG annotation, lipid-related DEGs genes were recruited from maize root transcriptome data. A total of 609 lipid-related genes were further classified into specific pathways. Figure 5 shows the number of genes involved in each lipid metabolism pathway. The results revealed that under saline–alkaline stress (100 mmol NaHCO_3_ treatment), various lipid-related pathways were stimulated (Figure 5). In the “24 h vs. 0 h” group, genes involved in the “eukaryotic galactolipid and sulfolipid synthesis,” “phospholipid signaling,” and “prokaryotic galactolipid, sulfolipid, and phospholipid synthesis” pathways exhibited significant up-regulation, with DEG distribution of 5-up/0-down and 8-up/2-down, 15-up/6-down and 67-up/50-down, and 7-up/1-down and 36-up/16-down (Log_2_FC ≥ 1.5 or ≤ -1.5), respectively. The differential expression of genes involved in the “sphingolipid biosynthesis” pathway was 8-up/5-down in “24 h vs. 0 h” and 32-up/23-down in “72 h vs. 0 h” (Log_2_FC ≥ 1.5 or ≤ -1.5) comparison groups. Genes involved in the “fatty acid synthesis” pathway were significantly down-regulated in both “24 h vs. 0 h” and “72 h vs. 0 h” groups, respectively. The transcriptional regulation of genes involved in the “eukaryotic phospholipid synthesis and editing” and “fatty acid elongation and wax biosynthesis” were different in both “24 h vs. 0 h” and “72 h vs. 0 h” groups, whereas the genes involved in “eukaryotic phospholipid synthesis and editing” were mostly down-regulated (16-up/28-down) in “72 h vs. 0 h” group. The DEGs in “fatty acid elongation and wax biosynthesis” were 35-up/9-down and 57-up/77-down in “24 h vs. 0 h” and “72 h vs. 0 h” groups, respectively. In the “triacylglycerol synthesis and degradation” and “oxylipin metabolism” pathway, a large number of genes were up-regulated under saline–alkaline stress. These results indicated that the transcriptions of genes involved in phospholipid and galactolipid metabolism pathways were activated under saline–alkaline stress.

### Analysis of Significant DEGs Involved in Lipid Metabolism Under Saline–Alkaline Stress

**FIGURE 6:**
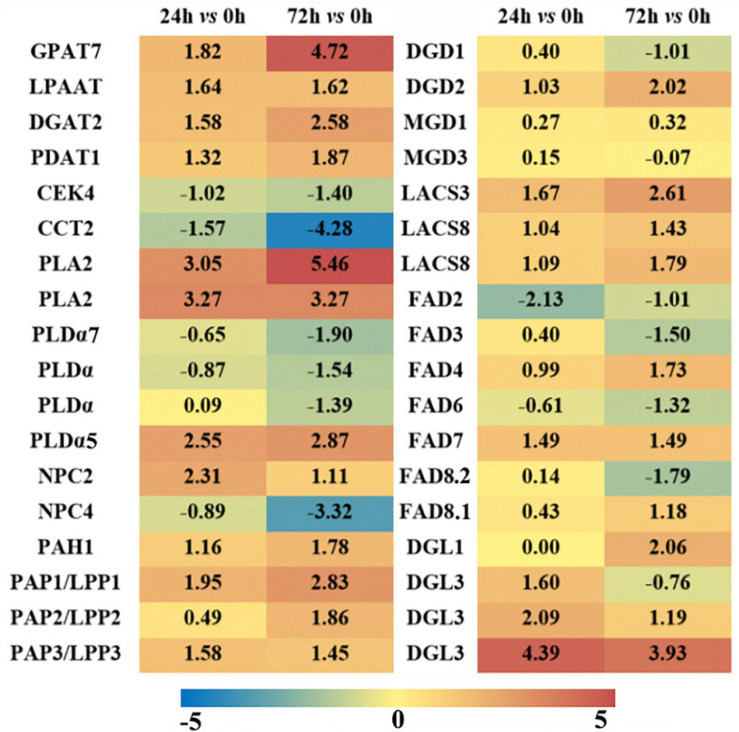
Differentially expressed genes (DEGs, Log_2_FC ≥ 1.5 or ≤ -1.5) involved in major lipid metabolism pathways in maize roots under NaHCO_3_ (100 mmol). A heatmap was constructed to illustrate the differential expression profiles of significant lipid related DEGs in “24 h vs. 0 h” and “72 h vs. 0 h” comparison groups. The number in each color block represents the Log_2_(fold change) of the corresponding genes, and the negative number represents down-regulated DEGs. The color scale was provided. Red color indicates higher expression level, and blue color indicates lower expression level.

As revealed in the previous part, it was evident that the metabolism of phospholipids and galactolipids was activated at the transcriptional level under saline–aline–alkaline stress. The expression profiles of a number of key genes involved in the major membrane lipid metabolism processes were illustrated in Figure 6 (see Supplementary Table S3 for a complete list). The *de novo* synthesis of triacylglycerol (TAG) in the ER is accomplished *via* three steps acylation of glycerol-3-phosphate (also known as the Kennedy pathway), which is also the necessary way leading to the synthesis of PC. Under saline–alkaline stress, genes involved in this pathway were apparently up-regulated, including some glycerol-3-phosphate acyltransferase (GPAT) and lysophosphatidyl acyltransferase (LPAAT) isoforms, and most of the diacylglycerol acyltransferase (DGAT) and phospholipids: diacylglycerol acyltransferase isoforms. It is worth noting that in another branch of the *de novo* synthesis of the PC (free choline to CDP choline), both the choline kinase and the choline phosphate cytidine transferase isoforms were down-regulated, which indicated that the *de novo* synthesis of PC was weakened.

PC and PE could be hydrolyzed by PLD and NPC to generate PA and DAG. In this study, three PLDs were up-regulated and three were down-regulated under 72 h saline–alkaline stress, and three NPCs were up-regulated, which means that both PLD and phospholipase C (PLC) pathways were partially triggered under saline–alkaline stress (Supplementary Table S3). The formation of PA and DAG *via* PA phosphatase (PAP)/lipid phosphate phosphatase (LPP) is a critical step for the synthesis of plastidic membrane lipids (MGDG and DGDG). Among the eight PAP/LPP orthologs, six putative membrane-bound and plastid-localized PAPs were induced by saline–alkaline (see Supplementary Table S3 for predicting transmembrane structure and subcellular localization). After 72 h of saline–alkaline stress treatment, the most highly induced LPP3 has a Log_2_FC of 3.67, which is an ortholog of AtLPPδ (AT3G58490). Another major pathway for PC degradation is mediated by phospholipase A (PLA) to form PLC. Two PLA2 genes were drastically up-regulated (Log_2_FC is from 3.05 to 5.46), which might be associated with the degradation of PC under saline–alkaline stress.

Monogalactosyldiglyceride synthase (MGD) and digalactosyldiglyceride synthase (DGD) are key enzymes responsible for the biosynthesis of plastidic galactolipids MGDG and DGDG. These genes were all up-regulated in the “24 h vs. 0 h” saline–alkaline stress treatment, but the changes were not significant. The up-regulation of *DGD2* was more notable at “72 h vs. 0 h” group, which has a Log_2_FC of 2.02. Fatty acid desaturase (FAD) is involved in the desaturation of phospholipids and galactolipids in plants (Welti et al., 2002), including PC acyl editing enzymes FAD2 and FAD3, PG acyl editing enzyme FAD4, and galactolipids desaturases FAD6, FAD7, and FAD8. Most of the genes encoding these FADs showed a slight induction in the “24 h vs. 0 h” group, whereas in the “72 h vs. 0 h” group, the plastidic FAD4, FAD7, and FAD8 were obviously up-regulated. The genes encoding a group of lipases that catalyze the hydrolysis of phospholipids and galactolipids to release free FAs were also up-regulated, including four diacylglycerol lipase (DGL) genes.

### Combined Analysis of Membrane Lipid Metabolic Changes in Maize Roots Under Saline–Alkaline Stress

**FIGURE 7:**
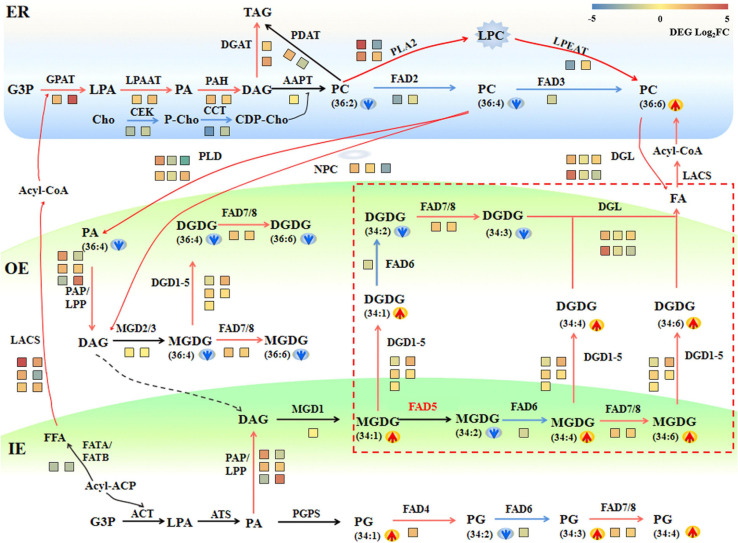
A schematic diagram of gene–metabolite network demonstrates lipid metabolism in maize roots under 72 h NaHCO_3_ (100 mmol). The glycerolipid synthesis pathways were depicted, and the involving genes and lipid metabolites were symbolized. The relative change of lipid molecular species was marked as arrows. Red and blue arrows indicate increased and decreased lipid metabolites, respectively. The relative expression levels of selected genes (72 h vs. 0 h) were marked as heat-map icons. The color scale of the heatmap was provided. Red color indicates higher expression level, and blue color indicates lower expression level. The red lines represent activated steps by NaHCO_3_ (100 mmol). ER, endoplasmic reticulum; OE, outer envelope. IE, inner envelope.

To interpret the regulation of metabolic changes of membrane lipids in maize roots under saline–alkaline stress, a combined analysis of transcriptome and lipidome was conducted, and a gene–metabolite network was constructed (Figure 7). In the ER compartment, genes encoding the main catalytic enzymes in the Kennedy pathway, such as GPAT, LPAAT, PAH, and DGAT, showed a significant up-regulation under saline–alkaline stress. PC is the most abundant phospholipids in maize root tissue, which is initially synthesized by transferring P-choline from CDP-choline to DAG in ER. The ER-generated PC is also an essential precursor to generate PA and DAG, through the hydrolyzing reaction mediated by PLD and/or NPC/DGK. Under saline–alkaline stress, a number of genes involved in PC degradation were obviously up-regulated, including PLD, NPC, and PAP. In addition, two PLA2 genes, mediating PC degradation to form PLC, were drastically up-regulated. These findings suggested enhanced PC turnover under saline–alkaline stress, which might explain the decreased PC content as revealed by lipidomic analysis.

As an important intermediate product of lipid metabolism and a bearer of lipid signals, PA can be produced in different subcellular through a variety of ways. In the inner plastid membrane, it is produced through a unique prokaryotic pathway and converted to DAG under the dephosphorylation of the plastid PAP. The transcriptomic data showed that three putative plastid-localized PAP/LPP genes were significantly up-regulated, which might correlate to the significant decrease in PA content. The galactolipid MGDG in the outer plastid membrane is produced by the MGD using DAG as the substrate, and DGDG could be produced from MGDG by DGD. In the transcription level, the synthesis of DGDG was partially activated through the up-regulated DGD2 under saline–alkaline stress. The expression of DGL genes, which converts glycerolipids (DGDG and PC) into free FAs, increased significantly, resulting in enhanced degradation of membrane lipids.

In plants, there are two pathways for the production of galactolipids: one is the eukaryotic pathway in the ER, and the other is the prokaryotic pathway in the plastid/chloroplast. Maize as an 18:3 plant, its galactolipid synthesis is completely dependent on the eukaryotic pathway, which is mainly reflected by the dominating C36:6 (two 18:3 acyl chains) molecular species in their galactolipids MGDG and DGDG. In this study, an increased level of C34:4, C34:5. C34:6 under saline–alkaline stress was observed, which implied that the saline–alkaline stress might be able to trigger the prokaryotic pathway to produce the C34 galactolipids.

### Transcription of Lipid Transport and Transcriptional Factors in Maize Roots Under Saline–Alkaline Stress

**FIGURE 8:**
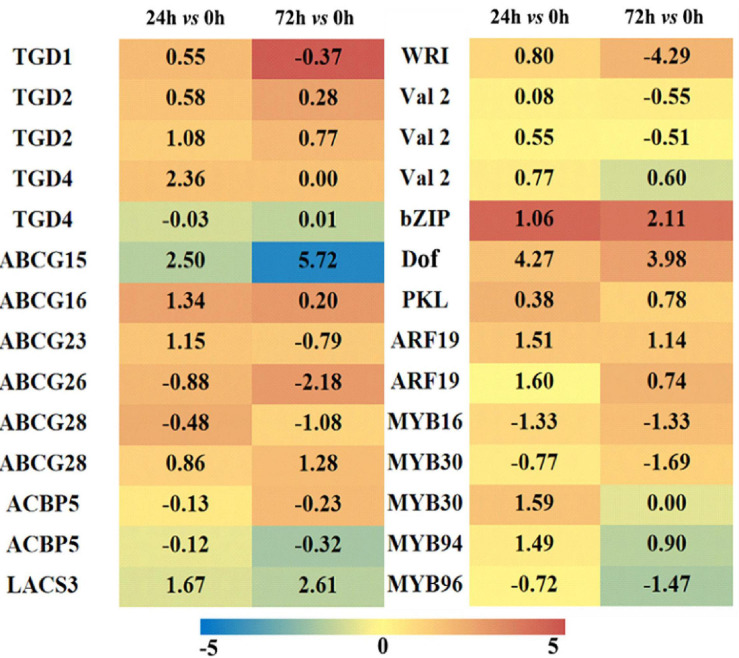
Heatmap of lipid-associated transporters and transcription factors differentially expressed in maize roots under NaHCO_3_ (100 mmol). A heatmap was constructed to illustrate the differential expression profiles of significant lipid related DEGs in “24 h vs. 0 h” and “72 h vs. 0 h” comparison groups. The number in each color block represents the Log_2_(fold change) of the corresponding genes, and the negative number represents down-regulated DEGs. The color scale was provided. Red color indicates higher expression level, and blue color indicates lower expression level.

The DEGs related to lipid transport and transcriptional factors were screened, and their differential expression profiles were presented by a heatmap (Figure 8, and the detailed information is on Supplementary Table S4). In plants, although the synthesis of FAs occurs in chloroplasts, the synthesis of different kinds of lipids occurs in different compartments, thus requiring lipid transport between different organelles (Li N. et al., 2016). In our RNA-seq data, most of the genes encoding trigalatosyldiacylglycerol proteins were up-regulated under saline–alkaline, but not significantly up-regulated. ATP-binding box is a type of protein that outputs lipids from the plasma membrane, and the most up-regulated ATP-binding cassette G transporters showed a Log_2_FC 5.72 in the “72 h vs. 0 h” group. In addition, two DEGs encoding Acyl-CoA binding protein (ACBP5), which associates with the transfer of FAs out of the plastid, were both down-regulated. Long-chain fatty acyl-CoA synthetase (LACS3) is associated with FAs and lipid transport, and the Log_2_FC of LACS3 was 1.67 and 2.61 in the “24 h vs. 0 h” and “72 h vs. 0 h” groups, respectively. These results suggested that the lipid transport among different organelles might be important for the lipid remodeling in maize roots under saline–alkaline stress.

In the transcriptome data, the DEGs encoding transcriptional factors were also screened out, and 48 transcriptional factor families were determined with a total of 674 transcriptional factors. The transcriptional factors closely related to lipid metabolism are demonstrated in Figure 8. The maize WRI1 was up-regulated in “24 h vs. 0 h” group and significantly down-regulated in “72 h vs. 0 h” group. This is consistent with our findings that a large number of genes were up-regulated during FA and TAG synthesis in the “24 h vs. 0 h” group, whereas some genes were down-regulated in the “72 h vs. 0 h” group. Other related transcriptional factors exhibited varied degrees of activated expression under saline–alkaline condition, particularly some members from bZIP and Dof families, which might be associated with the transcriptional regulation of FA and/or lipid metabolism under saline–alkaline stress.

## Discussion

As the most widely planted crop in the world, maize is sensitive to saline–alkaline stress, and its production is largely limited in the salinized areas. Salinity stress could cause changes in cell membrane permeability, resulting in membrane lipid structure and membrane protein damage, which affects the normal physiological function of the cell membrane (Greenway and Munns, 1980). The root tissues of crops directly contact with the soil thus would first sense the saline–alkaline stress. In this study, maize root tissues were used as the research materials, and combined transcriptomic and lipidomic analyses were conducted to investigate the regulation of membrane lipid metabolism under saline–alkaline stress.

Lipidomic analysis has played an important role in revealing the changes of membrane lipid metabolism in crops under various abiotic stresses, including wheat under low- and high-temperature stress (Li Q. et al., 2016; Narayanan et al., 2016) and maize under cold stress (Gu et al., 2017). There is only scarce information on the lipid metabolic changes in plant response to saline–alkaline stress (Guo et al., 2019). In this study, lipidomic analysis by UPLC/MS detected a large number of lipids including the major phospholipids, lysophospholipids, and plastidic galactolipids. In maize roots, PC was the most abundant lipid species, whereas the level of PC decreased significantly under saline–alkaline stress (100 mmol NaHCO_3_ treatment). As illustrated in the gene–metabolite network (Figure 8), PC degradation was enhanced through both PLA pathways, as evidenced by up-regulated PLA2 genes and increased PLC level, and the PLD and NPC/DGK pathway as supported by the up-regulated expression of a number of PLD and NPC isoforms. Our previous studies have revealed that the degradation of PC under low temperature conditions is mainly through the PLD pathway in maize leaf tissues (Gu et al., 2017). The hydrolyzation of PC mediated by PLD and NPC/DGK pathway produced PA and DAG, respectively, which are important intermediates in lipid metabolism (Canonne et al., 2011; Botella et al., 2017). In plants, PLD is directly hydrolyzed by phospholipid PC to produce PA, and NPC catalyzed reaction directly produces DAG (Hurlock et al., 2015; Li et al., 2015; Narayanan et al., 2016).

Although the plastidic galactolipids MGDG and DGDG are not dominating in membrane lipids in maize roots, they are important for maintaining the integrity of plastid membrane lipids (Narasimhan et al., 2013). In plant cells, there are two completely independent pathways for galactolipid synthesis, including the plastid/prokaryotic pathway and the ER/eukaryotic pathway (Frentzen et al., 1983; Browse et al., 1986). Plants that produce galactolipids through both prokaryotic and eukaryotic pathways are called 16:3 plants, which contain almost equal proportion of C34:6 and C36:6 in their MGDG and DGDG; plants that produce glycerolipids only through eukaryotic pathways are called 18:3 plants, which feature dominating C36:6 in their MGDG and DGDG (Ohlrogge and Browse, 1995; Li-Beisson et al., 2013). Our previous study revealed dominating C36:6 (two 18:3 acyl chains) molecular species in maize galactolipids (MGDG and DGDG); therefore, maize is proven to be a 18:3 plant whose synthesis of glycerolipids is almost entirely dependent on the eukaryotic pathway (Gu et al., 2017).

**FIGURE 9:**
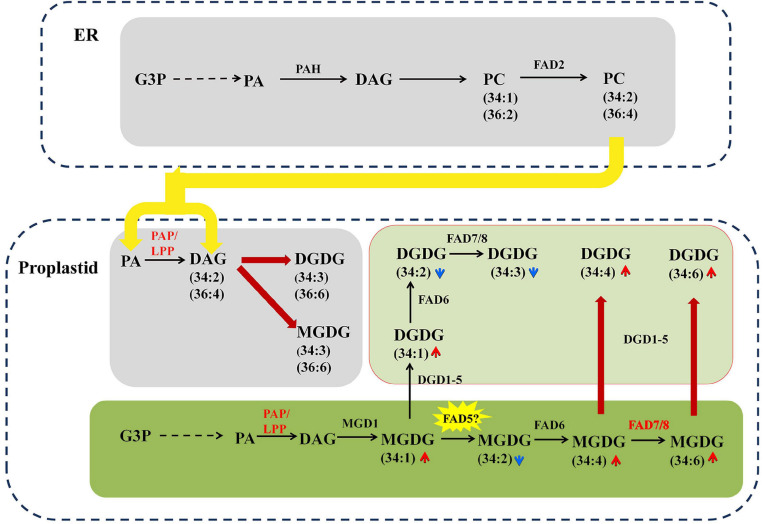
The proposed interaction and intermediates exchange between phospholipids pathway and galactolipid pathway in maize under NaHCO_3_ (100 mmol). The glycerolipid synthesis pathways were depicted, and the involving genes and lipid metabolites were symbolized. The relative change of lipid molecular species was marked as arrows. Red and blue arrows indicate increased and decreased lipid metabolites, respectively. The colored and bold lines represent activated steps by NaHCO_3_ (100 mmol).

These two pathways are synergistic, and the glycerolipid metabolism pathways between the ER eukaryotic pathway and the plastid prokaryotic pathway could be adjusted with changes in the external environment (Li et al., 2015). In this study, the galactolipids DGDG decreased significantly, and MGDG did not change a lot under saline–alkaline. DGDG has been reported to be a major chloroplast bilayer lipid and plays an important role in maintaining membrane integrity under stress (Li et al., 2015; Lin et al., 2016; Zheng et al., 2016). The significant decrease in DGDG might indicate the damaging effect of saline–alkaline stress on cell membrane. The parallel transcriptomic analysis revealed that a large number of the genes involved in the phospholipid and galactolipid pathways were significantly up-regulated, indicating the activated membrane lipid metabolism in maize roots under salinity condition (Figure 9).

It is worth noting that, under saline–alkaline stress, the number of C34 molecules, including that of C34:4, C34:5. C34:6, was found tremendously elevated (3–6-fold) in the galactolipids MGDG and DGDG. As maize has been proven to be a typical 18:3 plant, this new finding implied one possibility that saline–alkaline stress might be able to trigger the prokaryotic pathway to produce the C34 galactolipids. The low PAP enzyme activity in the chloroplast has been suggested to be the reason for the dysfunction of prokaryotic pathway in 18:3 plants (Frentzen et al., 1983; Heinz and Roughan, 1983). The formation of PA and DAG *via* PAP/LPP is a critical step for the synthesis of plastidic membrane lipids (MGDG and DGDG) (Eastmond et al., 2010). Previous research on the PAP family revealed two soluble PAHs, which had no transmembrane domain and were distinct from the non-soluble nature of their homologs AtPAH1&2 (Nakamura et al., 2007; Eastmond et al., 2010; Craddock et al., 2015). In *Arabidopsis*, in addition to the aforementioned soluble PAHs, there is a group of membrane-bound LPPs, some of which have been considered to be plastidic PAP (Pierrugues et al., 2001; Nakamura et al., 2009). In our transcriptome data, a total of 8 PAP/LPP orthologs were identified, and six of which represent membrane-bound and putative plastid-localized PAPs (see Supplementary Table S3 for predicting transmembrane structure and subcellular localization). Under saline–alkaline stress, three putative plastid-localized PAP/LPPs were significantly induced, and the most highly induced LPP3 had a Log_2_FC of 3.67, which is an ortholog of AtLPPδ (AT3G58490) (Nakamura et al., 2007). It appeared that the activated expression of PAP/LPP genes could partially restore the function of prokaryotic pathway in plastids, resulting the remarkable remodeling of membrane lipids in response to saline–alkaline stress.

FADs are involved in the desaturation of glycerolipids in plants (Welti et al., 2002). FAD5–8 catalyze the unsaturation of FA substrates esterified to plastidial lipids, which result in high linolenic (18:3) lipid species and are responsible for the generation of C34:6 molecules (Dunn et al., 2004). Under saline–alkaline stress, the increased expression of FAD7 and FAD8 was observed, which might be another contributor to the elevated level of C34:6 MGDG and DGDG (Figure 9). FAD5 required for the desaturation of C34:1 MGDG to C34:2 MGDG was not found in our transcriptome data, which may await further study.

In this study, the combined lipidomic and transcriptomic analysis revealed the activation of phospholipid and galactolipid metabolism pathways in response to saline–alkaline stress on both biochemical and transcriptional levels and the modular regulation of metabolite accumulation and gene expression. An increased level of C34 galactolipids under saline–alkaline stress implied that the saline–alkaline stress might be able to trigger the prokaryotic pathway. As the information on explicating the mechanism underlying the lipid regulation in18:3 plants has been lacking, this study shed a light on understanding the regulation of glycerolipid metabolism in 18:3 plants and for deciphering the roles of lipid remodeling in saline–alkaline stress response in major field crops.

## Data Availability Statement

The datasets presented in this study can be found in online repository SRA/NCBI (Sequence Read Archive/National Center for Biotechnology Information) with accession number SRP307694.

## Author Contributions

XX and JZ performed the experiments and prepared the manuscript. YW, SG, and YH prepared the samples. JZ, BY, JL, and ZL analyzed the data. JX and CZ conceived the experiments and revised the manuscript. All authors contributed to the article and approved the submitted version.

## Conflict of Interest

XX was employed by company Beijing Hortipolaris Co., Ltd. The remaining authors declare that the research was conducted in the absence of any commercial or financial relationships that could be construed as a potential conflict of interest.

## Footnotes

1 http://blast.ncbi.nlm.nih.gov/Blast.cgi

2 https://web.expasy.org/protscale/

3 https://wolfpsort.hgc.jp/

## Supplementary Material

Supplementary Figure 1 Quantitative RT-PCR verification of differentially expressed genes of roots of maize seedlings under NaHCO_3_ stress (100 mmol).

Supplementary Table 1 The original data of major lipids.

Supplementary Table 2 Differentially expressed genes in maize roots transcriptome under NaHCO_3_ (100 mmol).

Supplementary Table 3 The differentially expressed genes (DEGs, Log_2_FC ≥ 1.5 or ≤ -1.5) were further screened out from the total lipid related genes.

Supplementary Table 4 The differentially expressed lipid-related transporters and transcriptional factors in maize roots under NaHCO_3_ stress (100 mmol).

Supplementary Table 5 The subcellular localization and transmembrane structure prediction of related genes.

Supplementary Table 6 Primers used for quantitative real-time PCR.

## Abbreviations

DAG: Diacylglycerol
DGAT: diacylglycerol acyltransferase
DGD: digalactosyl diacylglycero synthase
DGDG: digalactosyldiacylglycerol
DGK: diacylglycerol kinase
DGL: diacylglycerol lipase
FAD: fatty acid desaturase
GPAT: glycerol-3-phosphate acyltransferase
LPAAT: lysophosphatidyl acyltransferase
LPP: lipid phosphate phosphatase
MGD: monogalactosyl diacylglycerol synthase
MGDG: monogalactosyldiacylglycerol
NPC: non-specific phospholipase C
PA: phosphatidic acid
PAH: phosphatidate phosphohydrolase
PAP: phosphatidic acid phosphatase
PC: phosphatidylcholine
PE: phosphatidylethanolamine
PG: phosphatidyl glycerol
PI: phosphatidylinositol
PLA: phospholipase A
PLC: phospholipase C
PLD: phospholipase D
PS: phosphatidylserine
SQDG: sulfoquinovosyl diacylglycerol
TAG: triacylglycerol.
