# Supplementary figures and images for: The Adjustment of Membrane Lipid Metabolism Pathways in Maize Roots Under Saline–Alkaline Stress
Source: Front Plant Sci. 2021 Mar 15;12:635327. doi: 10.3389/fpls.2021.635327 (PMC8006331; doi:10.3389/fpls.2021.635327)

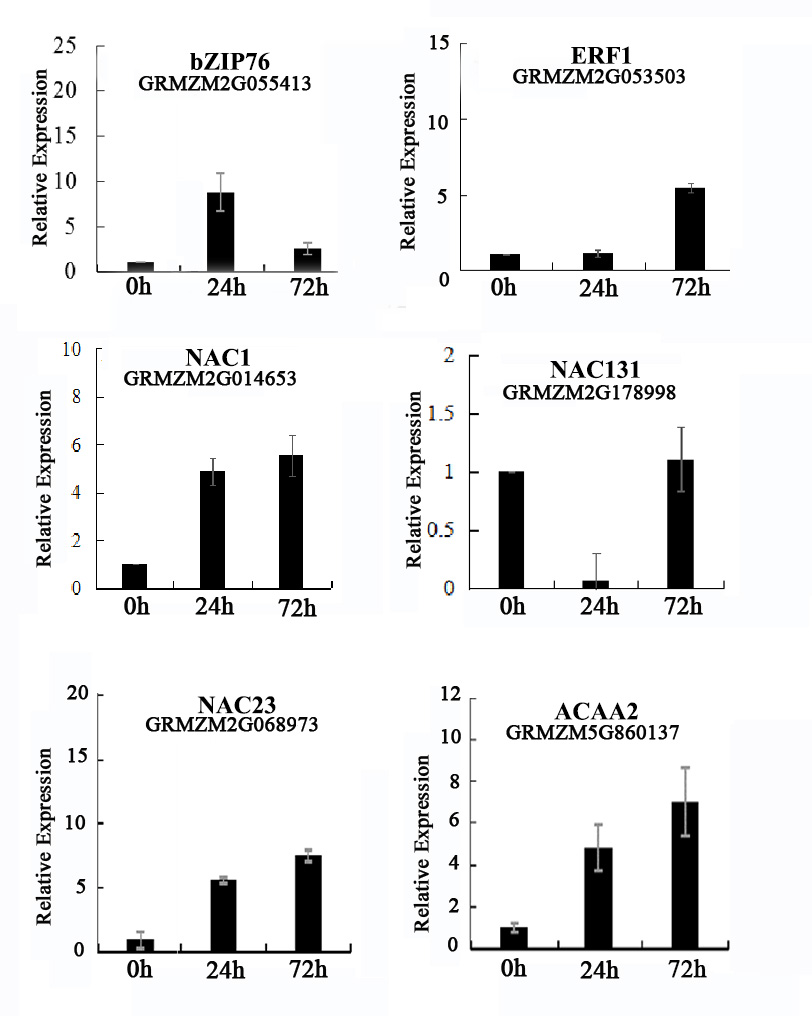

Supplement: Supplementary Figure 1 — Quantitative RT-PCR verification of differentially expressed genes of roots of maize seedlings under NaHCO3 stress (100 mmol). [file Image_1.JPEG]
